# Transcriptome data sets of free-living diplomonads, *Trepomonas* sp. and *Hexamita* sp.

**DOI:** 10.1128/MRA.00506-23

**Published:** 2023-11-01

**Authors:** Keitaro Kume, Tsubasa Gen, Kazutaka Abe, Hiroshi Komatsuzaki, Euki Yazaki, Goro Tanifuji, Ryoma Kamikawa, Yuji Inagaki, Tetsuo Hashimoto

**Affiliations:** 1 Institute of Medicine, University of Tsukuba, Ibaraki, Japan; 2 Graduate School of Science and Technology, University of Tsukuba, Ibaraki, Japan; 3 Graduate School of Comprehensive Human Sciences, University of Tsukuba, Ibaraki, Japan; 4 Graduate School of Life and Environmental Sciences, University of Tsukuba, Ibaraki, Japan; 5 Research Center for Advanced Analysis, National Agriculture and Food Research Organisation, Ibaraki, Japan; 6 RIKEN iTHEMS, Wako, Saitama, Japan; 7 Department of Zoology, National Museum of Nature and Science, Ibaraki, Japan; 8 Graduate School of Agriculture, Kyoto University, Kyoto, Japan; 9 Center for Computational Sciences, University of Tsukuba, Ibaraki, Japan; 10 Faculty of Life and Environmental Sciences, University of Tsukuba, Ibaraki, Japan; University of California, Riverside, California, USA

**Keywords:** evolutionary biology, parasitology, protists, bioinformatics, *Giardia*, *Trichomonas*

## Abstract

Most species belonging to the diplomonad genera, *Trepomonas* and *Hexamita*, are considered to have secondarily adapted to free-living lifestyles from the parasitic ancestor. Here, we report the annotated transcriptome data of *Trepomonas* sp. NIES-1444 and *Hexamita* sp. NIES-1440, the analysis of which will provide insights into the lifestyle transitions.

## ANNOUNCEMENT

Diplomonadida, a microaerophilic/anaerobic protist subgroup of Fornicata, comprises various parasitic/commensal genera. Interestingly, most species of the genera *Trepomonas* and *Hexamita* are considered to have secondarily adapted from parasitic to free-living lifestyles (1, 2). Since the sequence data of these species are crucial for understanding lifestyle transitions, we conducted RNA-seq analyses on *Trepomonas* sp. and *Hexamita* sp. and annotated these assemblies. We also annotated the existing transcriptome assembly of a free-living fornicate, *Kipferlia bialata* (3).

We obtained *Trepomonas* sp. NIES-1444 and *Hexamita* sp. NIES-1440 strains from the NIES collection. The cultures were maintained at 17°C in UYTS + rice medium (by ultrapure water; https://mcc.nies.go.jp/02medium-e.html#uyts_rice), and each protist was mass-cultured in flasks with fresh medium (500 mL) with its diverse food bacteria maintained in the culture medium for 1 week.

For RNA extraction, cells were collected by the following methods.

For *Hexamita* sp., density-gradient centrifugation was performed at 17°C, 800 × *g* for 20 min using the 0%, 10%, 20%, and 70% Optiprep (Sigma) gradient modified from the previous method (3). Purified cells were processed for RNA extraction with TRIzol and Turbo DNA-free Kit (Ambion), followed by PolyA selection using Dynabeads mRNA Purification Kit (Ambion). Following SMART-Seq v.4 Ultra Low Input RNA Kit for Sequencing (Illumina), we constructed libraries for RNA-seq analysis with Nextera XT DNA Library Prep Kit (Illumina) and analyzed these on a MiSeq (Illumina, 300-bp paired end).

For *Trepomonas* sp., cells were collected by two-round centrifugation at 4°C, first with 2,030 × *g* for 20 min, then 4,670 × *g* for 5 min. Using TRIzol, we extracted total RNA and provided 100 µg for Eurofins Genomics [polyA-selected RNA-seq, HiSeq2000 (Illumina), TruSeq SBS Kit v.3, 100-bp paired end].

We obtained 50,863,282 and 291,258,186 reads for *Hexamita* sp. and *Trepomonas* sp. Default parameters were used except where otherwise noted (see doi: 10.5281/zenodo.8246431). Reads were preprocessed using Trimmomatic v.0.39 [option: ILLUMINACLIP: (TruSeq3-PE-2.fa/NexteraPE-PE.fa) :3:30:10; LEADINGL: 30; TRAILING: 30; SLIDINGWINDOW: 4:25; MINLEN: 36] (4) and assembled using Trinity v.2.15.0 (option: max_memory: 200 G, CPU: 128) (5). ORFs (open reading frames) were determined for both assemblies using TransDecoder v.5.5.0 (https://github.com/TransDecoder/TransDecoder) (option: genetic_code: Hexamita, m: 50). We removed putative contaminants based on BLASTP (6, 7) against NCBI-nr v20221219 if they met all of the following criteria for the BLASTP top hit: *e* value of ≤1e-10, percentage of identical matches of ≥0.95, and query coverage per subject of ≥0.5, except when Metamonada NCBI: txid2611341 was hit in the hit list with an *e* value of ≤1e-10.

To compare the transcriptomes of these free-living diplomonads with another free-living fornicate, we also analyzed the Trinity assembly of *K. bialata* reported in the previous study (3). These three resultant assemblies were annotated by the best-hit annotation of InterProscan v.5.52 (8, 9) or BLASTP against the local database (Uniprot v.20230125 + *Trichomonas*/*Giardia*/*Trepomonas*/*Spironucleus* data: PRJNA16084/1439/288252/60811). If both BLASTP and InterProScan annotations were available, we used the former if its *e* value was ≤1e-40; otherwise, we chose the latter. Non-annotated CDSs (coding sequences) of ≥300 aa were added as hypothetical proteins. Final statistics, including assembly completeness (protein-mode BUSCO v.5.1.2) (10), are in Table 1.

**TABLE 1:** Overview of transcriptome sequences for the three fornicates

Organism		*Kipferlia bialata*	*Trepomonas* sp. NIES-1444	*Hexamita* sp. NIES-1440
Predominant bacterial genera (top 3) contaminated in culture (percentage of sequences per genus against all bacterial sequences)		*Pseudoalteromonas* (100)^*b*^	*Undibacterium* (38.9), *Chryseobacterium* (20.0), *Sulfurospirillum* (12.5)	*Cutibacterium* (85.5), *Ralstonia* (2.3), *Alcanivorax* (1.1)
No. of bases sequenced (Gb)		27.6^*b*^	29.1	7.6
No. of assembled transcripts		20,294^*b*^	76,247	48,794
No. of bases in assembled transcripts (kb)		28,897^*b*^	61,267	38,374
No. of annotated CDS regions		10,283	37,247	24,666
GC content of CDS regions (%)		56.9	43.1	45.9
N50 of CDS regions (bp)		2,820	1,466	1,295
Average length of annotated proteins (aa)		516.3	370.1	328.9
BUSCO completeness for annotated proteins (data set: eukaryote odb 10)	Complete^*a*^	33.4 (S:31.8, D:1.6)	20.4 (S:11.4, D:9.0)	15.3 (S:10.6, D:4.7)
	Fragment	12.2	7.5	9.0
	Missing	54.4	72.1	75.7
Reference	Accession no.	PRJDB5457 and DRR083618	PRJDB15716 and DRR460931	PRJDB15717 and DRR460932
	Publication^*b*^	Reference (3)	–	–

^*a*^ D, duplicate; S, single.

^*b*^ Numbers updated from original report; –, none, this study.

## Data Availability

BioProject numbers are PRJDB15716/PRJDB15717 (*Trepomonas*/*Hexamita*). Raw sequence and TSA (Transcriptome Shotgun Assembly) data are available at DDBJ/ENA/GenBank under accession numbers DRR460931/DRR460932 (*Trepomonas*/*Hexamita*) and ICTH01000001-ICTH01037247/ICTI01000001-ICTI01024666/ICTJ01000001-ICTJ01010283 (*Trepomonas*/*Hexamita*/*Kipferlia*).
